# Use of Portfolio-based Learning and Assessment in Community-based Field Curriculum

**DOI:** 10.4103/0970-0218.40873

**Published:** 2008-04

**Authors:** Swaroop Kumar Sahu, MB Soudarssanane, Gautam Roy, KC Premrajan, Sonali Sarkar

**Affiliations:** Jawaharlal Institute of Postgraduate Medical Education and Research, Pondicherry, India

**Keywords:** Portfolio, self-assessment, feedback

## Abstract

Portfolio-based learning is recognized in medical education. It helps students to assess themselves as per the key learning objectives and outcomes expected out of them. The faculty could also get feedback regarding individual student's progress toward learning outcomes and facilitate the students achieve the same. This article addresses the process of portfolio development and assesses from students feedbacks, if portfolio-based learning is an improvement over record-based study in community-based field studies. The results of this study shows that involving students in framing objectives, developing a mechanism for self-introspection and self-assessment by the students and a mechanism by which faculty can monitor each student's progress toward the defined objectives can significantly enhance the learnability of the students.

A portfolio is a purposeful collection of student work that exhibits the student's efforts, progress, and achievements in one or more areas. The collection includes student's participation in selecting contents, the criteria for selection, the criteria for judging merit, and evidence of student's self-reflection. A portfolio provides a comprehensive view of student performance. It is a portfolio where the student is a participant in, rather than the object of assessment. It provides a forum that encourages students to develop abilities needed to become independent, self-directed learners.(1) Portfolio-based learning is recognized to be useful in continuing medical education(2,3) because of the autonomy it gives to the adult learner.

In Department of P&SM, JIPMER, Pondicherry, students have Family Health Advisory Program (FHAP), where medical student from 4^th^ to 6^th^ semester of MBBS curriculum, in groups of five to six students, follow-up a family for three semesters on weekly basis. For years, we have been using record-based teaching learning process in FHAP. In the present new batch of 4^th^ semester, students we tried to initiate portfolio-based learning, in which, students were encouraged to set-up their own learning objectives with the help of teachers. The idea was that the portfolio-based learning would primarily address student's self-learning abilities and their attitude along with learning related to cognitive and psychomotor domain. This should also help students to assess themselves as per the key learning objectives and outcomes expected out of them and the faculty could also get feedback regarding individual student's progress toward the learning outcomes and facilitate the students achieve the same. Moreover, this type of learning was expected to shift the focus of students from record filling to problem-solving activities. This article addresses the process of portfolio development and assesses, from students feedbacks, if portfolio-based learning is an improvement over record-based study in community-based field studies.

## Materials and Methods

The students of fourth semester MBBS curriculum were asked to enlist the things that they felt worth learning in their FHAP. This exercise was done for the students at the beginning of their FHAP. This list was then analyzed into specific, valid, and measurable points. These points were used to define the broad areas of learning. After the broad areas of students interests were drawn, the more detailed points that the students wanted to learn under the broad headings were enlisted. These were discussed with all the faculty and residents of the department and the second draft of the broad categories of objectives and the minimum specific points that the students were expected to learn was drawn. This second draft was again discussed with students to finalize the seven areas that the students had to concentrate and the five specific sub-areas under each broad area that the students had to learn, practice, and impart health education in the respective families they were allotted.

The students were asked to identify problems in the family, they were following, from the 35 points that was finalized [Table 1]. They were also made free to identify any other issues, even beyond the enlisted 35 points, during their 18 months interaction with the family allotted to them. The given 35 points were just to guide students in identifying issues in the family, assist them to identify their learning objectives, give them the direction to initiate their self-learning, and to an extent standardize the program for all the groups of students. This 35-point issue list was also devised with the objective to improve the ownership of the program by the students. It was thought that if the students were asked to frame their own learning objectives, as they did at the beginning of the program, their ownership for the program had to increase.

**Table 1:** Identified 7 major areas and 35 sub-areas

Affective domain
Efficient in identifying the problems in family (listening skills, observation skills)
Effective communication
Initiativeness
Team-work
Empathy
Environment
Application of socio-economic status scales to the family being followed-up and give comments
Housing conditions - types of houses, overcrowding, latrines, hazardous condition
Social customs and its influence on health
Mental health - depression, dementia in elderly
Alcoholism and other addictions
Nutrition
Different methods of dietary survey (advantages and limitation of the various methods)
Dietary supplements
Foods rich/deficient in various vitamins and minerals and their tamil versions
Diet planning for a diabetic, hypertensive, pregnant/lactating mother
*Food fads and taboos*
Infant and children
Growth monitoring/interpretation of weight chart
Development of the infant
Breast feeding and weaning
Management and giving health education on common ailments such as respiratory tract infection, diarrhea, malnutrition, anemia, vitamin A deficiency, etc
Immunization
Family planning
Ideal duration of birth spacing
Ideal contraceptives for different situations
Common side-effects of the various contraceptives
Management of contraceptive failure
Decision-making process in adoption of contractive practice in the family
Ante-natal
Describe the health-seeking behavior from the time she got pregnant till return to house after delivery
Monitor pregnancy till delivery (including monitoring of routine investigations)
Describe their planning for the delivery (any extra personnel for managing at home and hospital, material/vehicle, money, etc)
Monitor post-natal care including number of post-natal visits
Describe any customs/functions done in family during pregnancy/after child birth
Tuberculosis
Disposal of sputum
Attitude of other members of the family toward the patient (isolation)
Treatment-seeking behavior before and after diagnosis and default management
Attitude of the patient toward DOTS agent and vice-versa
Follow-up (treatment, sputum examination)

The students were made clear that they were to address only the issues that they encounter in the family; they were not supposed to cover all the 35 enlisted issues in the family allotted to them. The students were expected to solve the problems that were present in the family by giving relevant advices to the family members.

At the time of giving health advice, they were asked to evaluate themselves regarding the quality of health education that they have imparted and identify their areas of deficiencies. The deficiencies thus identified had to act as a trigger for further self-learning to improve their own knowledge in the area and help them to impart better health education on the same topic in their subsequent visits to the family. Thus, they were asked to monitor the quality and effectiveness of their health education and also assess their strengths and weaknesses in imparting proper health education.

All the students may not encounter all the 35-points enlisted, in the family they were allotted, but they were encouraged to introspect how confident they would be in dealing with the situation if they had encountered such situations. These 35-point objectives thus helped the student to uniformly gather knowledge in all aspects they were expected to know, even if they do not encounter all the situations in the family they were following.

Self-evaluation of each student is an important aspect of any portfolio-based learning. These self-evaluations by the students in Table 2 were purely for the purpose of their self-introspection and they were clarified that the marks they award themselves would not be linked in any way to their internal assessment marks. This was important to ensure that the students do not unnecessarily inflate their self-assessment marks. These self-evaluations were done for every 2 months and these gave an opportunity to the guiding residents and faculty to see if the students were improving or not in any area and the reasons for the same. Thus, the residents and faculty could get an opportunity to facilitate learnability of students in their areas of weakness to achieve the desired objectives.

**Table 2:** Monthly self-evaluation form

	1	2	3	4	5	6
Affective domain						
Identifying problems						
Communication						
Initiativeness						
Team-work						
Empathy						
Environment						
SES scales						
Housing condition						
Social customs						
Mental health						
Addictions						
Nutrition						
Dietary survey						
Advantages and disadvantages						
Food rich in vitamins and minerals						
Diet planning						
Food fads and taboos						
Infant and children						
Growth monitoring						
Development						
Nutrition						
Common diseases						
Immunization						
Family planning						
Birth spacing						
Contraception						
Side-effects management						
Contraceptive failure						
Decision making						
Ante/post-natal						
Health-seeking behaviour						
High risk case						
From labor onset						
Post-natal care						
Customs/functions						
Tuberculosis						
Disposal of sputum						
Attitude						
Treatment seeking						
DOTS agent						
Follow-up						

At the end of two semesters, the students were asked to give feedback on a four/five point-likert scale [Table 3]. The feedback form contained seven questions; the students had to encircle the most appropriate choice that they felt was appropriate regarding the FHAP. This feedback was compared with the feedback taken from the previous batch of students who had completed their FHAP with record-based teaching. Anonymous feedbacks were taken from the students.

**Table 3:** Student feedback form

Please encircle the most appropriate choice that you feel is more appropriate regarding FHAP program
Objectives of FHAP were clear to you. To a great extent (1)/somewhat (2)/very little (3)/not at all (4) Was the FHAP posting useful to you? Very useful (1)/useful (2)/moderately useful (3)/of little use (4)/not useful (5) Was it useful to the family you were following? Very useful (1)/useful (2)/moderately useful (3)/of little use (4)/not useful (5) Participation of residents in guiding you was Excellent (1)/above average (2)/average (3)/below average (4)/extremely poor (5) Participation of faculty in guiding you was Excellent (1)/above average (2)/average (3)/below average (4)/extremely poor (5) How much you were interested in the program? To a great deal (1)/much (2)/some what (3)/little (4)/never (5) FHAP needs change in the approach of teaching. What is your opinion? Strongly agree (1)/agree (2)/undecided (3)/disagree (4)/strongly disagree (5)

The present batch of students also used the same records but the change in approach was that they were asked not to follow-up families page wise as given in records but to go into the family with an open mind, identify the problems in the family and give health education related to the identified issue. If they had any problem in giving health education when they first encountered it, that had to became a trigger point for self-learning so that they could give better advice in their subsequent home visits for that particular topic.

Among the 41 feedbacks from previous batch of students, 2 had submitted incomplete forms and of the 50 feedbacks got from the present batch of students, 1 had submitted incomplete form. Analysis was done using software package SPSS version 13.

## Results and Discussion

The results in Table 4 show that there was significant difference between the feedbacks given by students of previous batch and the present batch to the questions - if objectives of FHAP were clear to them, was the FHAP posting useful to them and was it useful to the family they were following. There was no statistical significant difference between the feedback given by students to the questions - regarding how much they were interested in the program, participation of faculty in guiding them, participation of the residents in guiding them, and their opinion regarding if FHAP needed change in the approach of teaching.

**Table 4:** Student feedbacks: Present and previous batch

	1	2	3	4	5	Mean (standard deviation)	MW test^2^ sig. (two-tailed)
Objectives of program were clear to you							
Previous (41)	3	19	13	6		2.54 (0.84)	0.000
Present (50)	6	41	2	1		1.96 (0.493)	
Program was useful to you							
Previous (41)	2	6	16	12	5	3.29 (1.031)	0.043
Present (50)	3	17	16	12	2	2.86 (0.99)	
Was useful to the family you were following							
Previous (40)	1	8	4	15	12	3.73 (1.176)	0.000
Present (49)	4	16	18	9	2	2.78 (0.985)	
How much you were interested in the program							
Previous (40)	6	8	14	10	2	2.85 (1.122)	0.355
Present (50)	6	15	21	5	3	2.68 (1.019)	
Program needed change in approach of teaching							
Previous (41)	22	14	2	0	3	1.73 (1.096)	0.193
Present (50)	20	20	6	3	1	1.9 (0.974)	
Participation of residents in guiding you							
Previous (41)	9	13	11	6	2	2.49 (1.143)	0.781
Present (50)	8	16	19	7	0	2.5 (0.931)	
Participation of faculty in guiding you							
Previous (41)	5	11	14	7	4	2.85 (1.152)	0.385
Present (50)	9	9	27	2	3	2.62 (1.028)	

MW test^2^: Mann-Whitney test

The results reflect that with the same amount of efforts from faculty and residents in both the batches, the objectives of FHAP was significantly better with the present approach as compared with the record-based approach alone. With the present approach, the student's feelings regarding the usefulness of the program for themselves and to the family they were following was significantly better when compared with the previous approach. But there was no significant difference in the interest levels between the previous and present batch of students. There was also no difference in the response between the batches to the question - “FHAP need change in the approach of teaching. What is your opinion?” May be this question is too broad to capture any difference between the perceptions of both the batches; the batch-wise difference between the reasons for opting for change in approach of teaching in FHAP would have reflected better picture. This is a limitation while taking feedback in the present study.

## Conclusion

To conclude, the results show that involving students in framing objectives, a mechanism for self-introspection and self-assessment by the students and a mechanism by which faculty can monitor each student's progress toward the pre-defined learning objectives can significantly enhance the learnability of the students.
